# VDAC—A Primal Perspective

**DOI:** 10.3390/ijms22041685

**Published:** 2021-02-08

**Authors:** Carmen A. Mannella

**Affiliations:** Department of Physiology, Center for Biomedical Engineering and Technology, University of Maryland School of Medicine, Baltimore, MD 20201, USA; mannellac@gmail.com

**Keywords:** mitochondria, VDAC, chemiosmosis, endosymbiosis, evolution, membrane transport, porin

## Abstract

The evolution of the eukaryotic cell from the primal endosymbiotic event involved a complex series of adaptations driven primarily by energy optimization. Transfer of genes from endosymbiont to host and concomitant expansion (by infolding) of the endosymbiont’s chemiosmotic membrane greatly increased output of adenosine triphosphate (ATP) and placed selective pressure on the membrane at the host–endosymbiont interface to sustain the energy advantage. It is hypothesized that critical functions at this interface (metabolite exchange, polypeptide import, barrier integrity to proteins and DNA) were managed by a precursor β-barrel protein (“pβB”) from which the voltage-dependent anion-selective channel (VDAC) descended. VDAC’s role as hub for disparate and increasingly complex processes suggests an adaptability that likely springs from a feature inherited from pβB, retained because of important advantages conferred. It is proposed that this property is the remarkable structural flexibility evidenced in VDAC’s gating mechanism, a possible origin of which is discussed.

## 1. Introduction

Life as we know it was made possible by chemiosmosis, by which the free energy of oxidation of food molecules is used to pump protons across a membrane [1]. The resulting chemiosmotic potential can be harnessed to do work, from rotating a flagellum to catalyzing the production of adenosine triphosphate, ATP, the biosphere’s energy currency. Maximizing the output of the latter process drove the endosymbiotic event that culminated in the evolution of the eukaryote (described in [2]). Of course, the conversion of the bacterial endosymbiont to the organelle that we now call the mitochondrion was not a single event but rather a complex series of molecular adaptations of both host and guest that are still being defined (e.g., [3,4]). This overview focuses on one of those adaptations, the conversion of a simple sieve protein to a central player in a sophisticated network of cell processes, made possible by a remarkable structural flexibility inherited from its endosymbiont precursor.

## 2. Folding the Chemiosmotic Membrane

A key change in the endosymbiont during its transition to proto-mitochondrion was the extensive infolding of its plasma membrane. By increasing the ratio of chemiosmotic membrane area to cell volume occupied, ATP output was greatly enhanced while leaving room for the other innovations of the eukaryotic lifestyle. Boosting energy production likely spurred extreme gene shedding by the endosymbiont, the adaptation that differentiated it from mere intracellular parasites. Outsourcing genes and associated protein production to the host freed up space (otherwise occupied by ribosomes) for expanding the ATP-generating membrane, as well as for enriching the soluble enzymes of energy metabolism. The alphaproteobacterium that became the endosymbiont already had the basic machinery (in particular, Mic60) for invaginating its plasma membrane [5,6]. However, this was a shadow of the complex mechanisms that would evolve to generate and remodel the mitochondrion’s cristae (reviewed in [7,8,9]), which can attain a surface area ten times or more that of the peripheral region of the membrane. The multiprotein complexes of the respiratory chain are easily recognizable between bacteria and mitochondria, but eukaryotic versions usually have more subunits with added functionality. A prime example is the ancient protein complex that generates ATP itself, the F_1_F_0_ ATP synthase. Although the fundamental process by which this nanomachine couples transmembrane potential to ADP phosphorylation is highly conserved, eukaryotic versions have an added feature: the ability to form dimers that bend—or stabilize the bends in—the expansive inner membrane (described in [10,11]). Clearly, the energy advantage of folding the chemiosmotic membrane underpinned many of the adaptations by which the endosymbiont became the mitochondrion.

## 3. Communication at the Host–Endosymbiont Interface

The mitochondrion is composed of two membranes, with the convoluted inner membrane encased within a morphologically simpler outer membrane. The latter likely derived from the endosymbiont’s outer envelope, retained and adapted to protect its all-important ATP-generating plasma membrane from external assault (e.g., by proteases, nucleases, lipases) while containing critical soluble proteins (such as kinases and cytochrome c) and maintaining a steady exchange of select macromolecules, metabolites, and ions with the host cytoplasm. In mitochondria, the critically important properties of interface integrity and permeability are primarily mediated by two proteins, VDAC and Tom40, that share a folding motif unique in nature, a 19-strand β-barrel.

### 3.1. Metabolite Transport

VDAC is generally recognized as the prime conduit for metabolite transport across the mitochondrion’s outer membrane. The acronym derives from the *v*oltage-*d*ependent partial closures and modest *a*nion selectivity of the large *c*hannel or pore formed by this ~30 kDa protein in planar lipid bilayers, properties that are conserved across the eukaryotic kingdoms [12,13]. VDAC comes in three isoforms, the two most common in higher eukaryotes (VDAC1 and VDAC2) having diverged from the oldest (VDAC3) around 300 million years ago [14,15,16]. None of the extant VDAC genes have an obvious direct ancestor among the bacterial porins, families of β-barrel proteins in bacterial outer envelopes that generally display greater selectivity and lower permeability than VDAC [15,17]. Thus, it is likely that the selection of a single β-barrel protein to regulate the permeability of the host–endosymbiont interface was an early event, one that coincided with internalizing the inner membrane.

Steady-state reaction rates of mitochondrial chemiosmosis are large, typically hundreds of molecules per minute per milligram of mitochondrial protein, equivalent to fluxes tens of molecules and ions per millisecond per square micrometer of inner-membrane surface. To support these reaction rates, the outer membrane, with only 10–30% the area of the inner membrane, must allow influx and efflux of hundreds of molecules and ions per millisecond per square micrometer of its surface. VDAC pores occur in large density on the outer membrane surface, 10^3^–10^4^ per square micrometer [18], with each pore capable of supporting the diffusion of 10^6^ ions and 10^2^–10^3^ metabolites per millisecond [17,19]. Thus, VDAC, in its open state (as defined by its conductance in bilayers) appears to confer on the outer membrane more than sufficient permeability (conservatively a 10- to 100-fold excess) to retain the energetic advantage of folding the inner membrane. At the same time, the bore of VDAC’s β-barrel (2.5–3 nm) is small enough to prevent exchange of soluble proteins between mitochondrial and cytosolic compartments [20].

### 3.2. Protein Import

The outsourcing of the endosymbiont’s protein production to the host necessitated another major adaptation, development of the protein import machinery named TOM and TIM, for *t*ranslocases of the *o*uter and *i*nner *m*embranes [21,22,23,24]. The latter coordinates with machinery in the matrix to assemble respiratory chain complexes, which include the few proteins whose genes were retained on mitochondrial DNA and translated on mitochondrial ribosomes. The central component of the TOM complex is Tom40, the other 19-strand β-barrel transport protein in the outer membrane. A possible scenario, suggested by phylogenetic and structural evidence [15,22], is that Tom40 and VDAC derived from a common precursor β-barrel protein (“pβB”). The primary task of the putative ancestral pore may well have been to transport not metabolites but unfolded polypeptides from the host cytoplasm. After all, the endosymbiont could not export its genes to the host unless it could, in turn, repatriate the associated proteins. The novel task may have necessitated a structural innovation, the 19-strand β-barrel, with sufficient collateral leakiness to small solutes to support the reactions of chemiosmosis in the endosymbiont. The complementary roles of pβB may be echoed in the properties of its descendents, with the mitochondrial polypeptide translocase pore Tom40 able to transport nicotinamide adenine dinucleotide (NADH) in VDAC-less yeast mutants [25] and the metabolite pore VDAC able to translocate α-synuclein into mitochondria [26].

The evolution of pβB into two functionally discrete proteins that optimize energy production via different processes took two very different paths. Proto-Tom40 locked into protein partnerships to form the TOM complex, conferring selectivity and efficiency on the import of proteins needed to build the energy transduction machinery of the inner membrane. Proto-VDAC, on the other hand, remained a solo player at the interface, with natural selection optimizing its permeability for metabolites critical to energy output [17] and, in the process, supplanting the other porins in the endosymbiont’s outer envelope. Additionally, proto-VDAC began to develop its “hub” personality, forming transient partnerships with other proteins either to regulate energy output (by altering its own permeability) or to participate in newly evolving, uniquely eukaryotic processes.

## 4. VDAC Regulates Outer Membrane Integrity

In eukaryotes, rupture of the mitochondrial outer membrane is generally a terminal event, associated with irreversible loss of chemiosmotic function and cell death (discussed in [27]). Generally, the outer membrane does not lyse directly (for example, in response to osmotic changes in the cytosol) but indirectly, by mechanical strain caused by contact with the inner membrane as it unfolds during matrix swelling. This can be a deliberate process, part of the cell’s quality control program, but is also associated with pathological events initiated by a variety of stresses and chemical insults. The process of mitochondrial membrane herniation during programmed cell death has been visually captured in a recent elegant study [28].

The mitochondrion’s outer membrane is remarkably resistant to osmotic stress thanks to the extreme permeability to small solutes conferred on it by VDAC [27]. In vitro, the outer membrane survives osmotic shocks ten-fold greater than needed to lyse liposomes or mitochondrial inner membrane vesicles of similar size. Given the apparent excess of VDAC in the outer membrane from the perspective of metabolite transport alone, the high surface density of the pore may have as much (or more) to do with preserving mitochondrial integrity by maintaining extreme osmotic inactivity of the interface. This has interesting implications when considering the selective pressures on the pβB protein. Regulating the integrity of the barrier membrane between host and endosymbiont would have controlled the rate of release of the latter’s DNA, central to the process of gene transfer to the host. Excessive DNA mixing in the host cytoplasm might have muddled this evolutionarily critical process, especially prior to development of the nucleus. In fact, there is evidence that VDAC can directly mediate permeation of DNA through membranes [29], related to its tendency to form oligomers in the outer membrane [30]. Controlled release of endosymbiont DNA (through an intact outer envelope) may have been another critical function of pβB.

Fast forward a billion or so years and the descendants of the endosymbiont now have integrated thoroughly into the metabolic and signaling pathways of eukaryotes, including those for fundamental processes like programmed cell death. Triggers of VDAC closure like tubulin dimers and glutamate become elevated early in apoptosis, suggesting that VDAC closure could be a deliberate tactic (alone or in conjunction with other mechanisms, such as aggregation of Bcl-2 family proteins) to induce outer membrane damage, by making the membrane more susceptible to minor stresses (discussed in [31]; also see Section 5.3).

## 5. VDAC Structure and Gating

**Figure 1:**
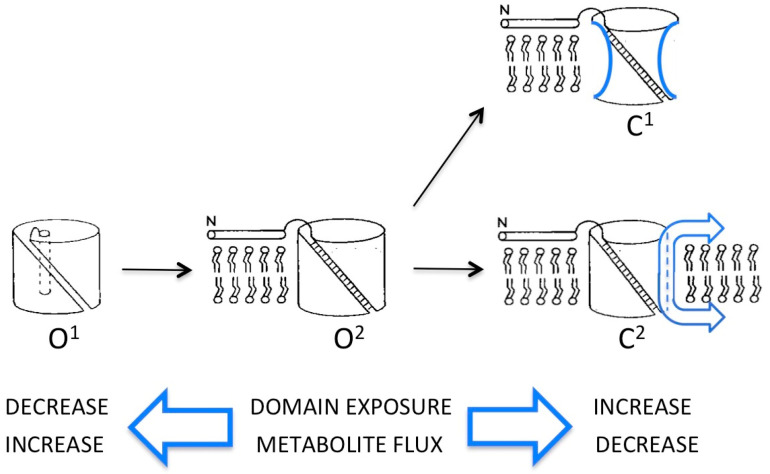
Schematic of open (O) and closed (C) conformers of the voltage-dependent anion-selective channel, VDAC, with the β-barrel represented as an open cylinder and the N-terminal α-helix as a narrow rod either inserted inside (O^1^) or extended away from (O^2^ and closed states) the pore lumen. Evidence in support of each conformer is described in the text. In C**^1^,** the sides of the cylinder are concave to signify narrowing of the β-barrel due to partial collapse of the walls. In C^2^, the cylinder has a smaller diameter due to removal (sliding) of segments of the VDAC polypeptide (represented as arrows) from the wall of the β-barrel to either membrane surface (see [17]).

Atomic structures of the mammalian VDAC protein in various lipid and detergent environments have revealed a common 19-strand β-barrel motif referenced above (represented schematically as O^1^ in Figure 1). The projected density of these structures (obtained by X-ray and NMR methods [32,33,34]) closely matched that of the native VDAC pore in the fungal membrane (obtained by cryo-electron microscopy of ordered arrays) with an internal pore diameter (2.5–3 nm) consistent with the permeability of “open” VDAC [35]. All this strongly suggested that the 19-strand β-barrel is a “physiological” structure—but perhaps not the only fold that the protein can assume. An alternative structure has been deduced from extensive electrophysiological studies of VDAC, reconstituted from detergent extracts into planar phospholipid bilayers (reviewed in [17]). The “bilayer” model is a somewhat smaller β-barrel comprised of 14 transmembrane strands including, somewhat problematically, the N-terminal α-helix. In the atomic structures, the N-terminal segment resides inside the β-barrel, secured against the barrel wall by salt bridges. In ordered arrays of VDAC in the outer membrane, the N-terminal region extends outside the pore lumen (state O^2^ in Figure 1).

### 5.1. Gating Is Intrinsic to VDAC

VDAC was discovered in extracts of *Paramecium* mitochondria by virtue of its pronounced gating characteristics: large step-wise drops in conductance of planar bilayers in response to relatively small transmembrane potentials (typically 40 mV) [36]. In fact, there are multiple subconductance states, the most common about half that of the “open” state (roughly 2 nS vs. 4 nS in 1M KCl). From the perspective of impact on chemiosmosis and ATP output, the permeability of the “closed” state to metabolites is greatly reduced, with ATP basically impermeant [19,37]. Several gating “effectors” were identified that increase or decrease the sensitivity of VDAC to voltage, including NADH, certain anionic polymers, and an as yet unidentified modulator protein that resides in the space between the outer and inner membrane (the intermembrane space).

The term “gating” is typically associated with blocking a pore opening (by mobile elements or ligands) or subtle rearrangements of its internal structure. The inhibition of VDAC permeability by tubulin is attributed to its (voltage-dependent) binding and physical obstruction of the pore opening [38]. However, the functional changes in VDAC induced by membrane potential appear to correlate with major conformational changes in the pore itself. Electrophysiological data suggest gating involves removal of multiple staves (β-strands and the N-terminal segment) from the conductance path, based on changes in ion selectivity caused by point mutations (reviewed in [17]). Reduced ion conductivity suggests narrowing of the inner diameter of the pore to ~1 nm. Involvement of a major structural change in gating was confirmed by the response of VDAC in planar bilayers to oncotic pressure, i.e., osmotic stress induced by large concentrations of impermeant polymers [39]. The observed decrease in internal volume of the pore caused by dehydration was consistent with the kind of large-scale structure shifts inferred from the electrophysiological data. Moreover, the response of the pore protein to osmotic stresses suggested that VDAC might be involved in sensing cytosolic and intramitochondrial colloidal pressure [40].

### 5.2. VDAC Occurs as an Ensemble of Conformers

Experiments were done that employed probes like proteases and monoclonal antibodies directed against specific epitopes to determine the topology of VDAC in situ, i.e., in the outer membrane of mitochondria in test tubes and in cells, as opposed to reconstituted from detergent into bilayers or other synthetic systems [41,42,43,44]. Results were generally consistent with atomic models (delineated in [43]), but confirmed that, in at least a fraction of VDAC proteins, there are domains buried in the atomic structure that are, in fact, accessible to the large probes. These include the N-terminal α-helix and three transmembrane β-strands, one at the C-terminus and two spanning residues 189-209. Exposure of the internal domain is consistent with the “bilayer” model for VDAC, which assigns it to a large external loop in the open state, while the terminal domains are proposed to move from inside the pore to the membrane surface during closure (represented as conformer C^2^ in Figure 1). Osmotic swelling of mitochondria enhanced accessibility of these VDAC regions to the macromolecular probes, attributed to their residing on the internal (inner-membrane-facing) surface of the outer membrane, accessible after its rupture. However, exposure of some “latent” VDAC domains was significant even in intact mitochondria, suggesting their accessibility at both OM surfaces, as postulated for VDAC gating in response to potentials of opposite polarity [17,19]. Interestingly, antibodies directed against the N-terminus tend to occur in clusters on the outer membrane, e.g., on narrow neck-like regions between adjacent heart mitochondria [45]. This strongly suggests that mechanisms that expose this domain and/or stabilize the corresponding conformers are cooperative, i.e., involve interactions between VDAC proteins.

A technical hurdle in these kinds of experiments is unambiguously locking the pore into defined functional states, which makes it difficult to resolve controversies. Absent such control, the most straightforward interpretation of existing results is that VDAC is a highly flexible polypeptide present as an ensemble of conformational states in the mitochondrion’s outer membrane, consistent with the multiplicity of conductance states (“open” and “closed”) observed in planar lipid bilayers [17,46,47] (see Figure 1). The atomic structures seen in micelles represent a low-energy “open” state (O^1^ in Figure 1) in which almost the entire polypeptide is folded into the 19-strand β-barrel, with N-terminal α-helix inside the pore lumen. Conversely, in planar VDAC arrays in the mitochondrial outer membrane, conformers clearly exist in which the N-terminal domain is outside the β-barrel. This conformation (O^2^ in Figure 1) was proposed to represent a metastable “open” state of the pore on the pathway to closure, stabilized in the membrane arrays by lattice forces [18]. Evidence for “oligomerization” of VDAC associated, for example, with translocation of unfolded polymers (like DNA) may have more to do with stabilizing the O^2^ conformer (which has the widest bore) than creating “megapores”. The “bilayer” model for VDAC with fewer β-strands in the pore wall, inferred from electrophysiological data, could represent a hybrid or intermediate “open” state, preferred in planar phospholipid membranes, with N-terminal inside and several β-strands moved to the surface.

The availability of atomic structures of VDAC has opened the door for computer simulations to predict permeability properties of different models (in particular, for ATP), and to propose models for “closed” states that, thus far, have eluded structure determination (reviewed in [48,49]). Computer modeling has confirmed the stabilizing role of the N-terminal segment inside the VDAC lumen and suggested an alternative mechanism for large-scale closure: internal collapse of the “floppy” pore wall [50] (conformer C^1^ in Figure 1). While feasible, this mechanism would not explain the observed accessibility to topological probes of VDAC domains (for example at the N- and C-termini) that are buried in the atomic models. If VDAC is as structurally variable as results suggest, solving the structure of “closed” conformers will likely require approaches like single particle cryo-EM, in which images of thousands of structurally heterogeneous examples of the same protein are sorted and averaged [51]. The small size of the VDAC protein combined with its extreme variability presents challenges that will require clever chemistry and molecular engineering to overcome, e.g., locking the protein into defined states and/or attaching it to partner proteins (including itself).

### 5.3. VDAC Gating Is a Stress Sensor

The aqueous compartment between the mitochondrion’s outer and inner membranes, the “intermembrane space” (IMS), has two distinctly different regions. The peripheral zone, in which the two membranes are adjacent (typically separated by only 10–20 nm), connects through narrow (20–40 nm) junctions to the space inside each crista, collectively called the intracristal space. There is growing evidence that the size and shape of cristae and their junctions are regulated to optimize the mitochondrion’s energy generating function, as reviewed in [27]. Likewise, there is considerable evidence that partitioning of ATP and ADP between the IMS and cytosol (mediated by VDAC) has an important regulatory effect on chemiosmosis, e.g., [52,53]. In addition, VDAC may play a critical role in maintaining mitochondrial ATP generation by sensing and adjusting to otherwise lethal internal stresses.

Volume changes in the mitochondrial matrix cause reciprocal changes in the intermembrane space. In mitochondria with numerous cristae (such as found in cardiac muscle), the IMS constitutes only ~10% of the total volume enclosed by the outer membrane (OM) [54]. Gradual matrix swelling rapidly collapses the intermembrane space, thereby dramatically increasing both oncotic pressure and the effective concentration of the VDAC modulator. Both effects of inner membrane expansion favor VDAC closure and reduced OM permeability to metabolites, which in turn could attenuate chemiosmosis and slow matrix swelling by processes that are energy dependent, such as minor imbalances in K^+^ fluxes [55]. In addition, coordinated VDAC gating by an internal signal (in this case, IMS collapse) would simultaneously signal to the cell that OM rupture may be imminent, by exposing previously “buried” VDAC domains for interactions with cytosolic partners (Figure 1).

While VDAC closure might protect mitochondria against low-level (physiological) adjustments in matrix volume, it would have no effect on matrix expansion associated with uptake of pathological levels of Ca^+2^ [55]. Large-scale mitochondrial swelling caused by the so-called permeability transition is associated with loss of inner membrane integrity and chemiosmotic function, and so would be largely unaffected by shutting off metabolite exchange at the outer membrane. Note, also, that a role for VDAC closure in preserving outer-membrane integrity in the face of routine stresses (such as slow matrix swelling) would not contradict its proposed involvement in deliberate OM rupture during apoptosis (Section 4). While increasing the osmotic sensitivity of the outer membrane (by VDAC closure) raises the risk of its rupture by cytosolic osmotic stresses, it pales in comparison to the imminent risk of membrane herniation from undeterred matrix expansion.

## 6. Possible Origin of VDAC’s Flexibility

It seems likely that the mechanism of VDAC gating has its origins in some idiosyncratic feature of pβB that was selected for as the host–symbiont relationship developed. The advantages conferred by this feature were sufficient for it to be retained by the proto-mitochondrion and passed on to all eukaryotes. Recent computational studies suggest that feature might be inherent instability of 19-strand β–barrels like those found in VDAC [56], reflected in the low energy barriers between its different subconductance states [17].

At first glance, there is an interesting parallel between mechanisms proposed for VDAC opening/closing [17] and for membrane insertion of β-barrel proteins, i.e., lateral sliding of adjacent strands in the wall of the barrel, e.g., [57]. In the latter process, the unfolded precursor protein interacts with the wall of another β-barrel (Sam50) to form β-hairpins prior to release into the membrane. In planar lipid bilayers, VDAC auto-inserts, with already formed β–barrel channels catalyzing the formation of new channels [58]. Might VDAC closure simply be reversal of the final steps in its membrane insertion, successively exposing domains by lateral sliding of β-hairpins out of the barrel wall, facilitated by stimuli that destabilize interactions with the N-terminal α-helix and mediated, perhaps, by interactions between VDAC proteins (Section 4)? In this scenario, pore opening would recapitulate the final stages of insertion, with β-hairpins sliding back into the barrel wall, possibly via interaction with adjacent VDAC molecules (assuming the role of Sam50) and locked in place by interaction with the N-terminal segment. If true, the conformational changes in pβB needed to regulate the interface between host and endosymbiont did not have to be invented. Rather, insertion of pβB simply needed to be fine-tuned (over eons) to allow partial reversal by useful stimuli.

## 7. Conclusions

Clearly, the results from electrophysiological and topological studies of VDAC suggest a protein that is amazingly agile, able to switch between conformers that vary both in permeability to metabolites and exposure of domains that, in turn, may interact with protein partners on either side of the OM interface (Figure 1). Structural and computational biology are providing important insights into VDAC’s molecular architecture and the permeability properties of some conformers, but have not yet caught up with the full range of conformational states that must exist in the native membrane. Whatever its origins, the remarkable structural flexibility of VDAC, likely inherited from a precursor 19-strand β–barrel in the endosymbiont, undoubtedly was central to its role in the evolving complexity of eukaryotes. Likewise, the protein’s ability to respond to signals (such as stresses) by conformational switching and to interact with new partners has been critical for the integration of mitochondria into additional metabolic and signaling pathways. However, throughout these important adaptations, VDAC has persisted as a pore with the primary mandate to support ATP production and regulate mitochondrial integrity.

## Data Availability

No new data were created or analyzed in this study. Data sharing not applicable to this article.
